# Supplementary material for: FERN – a Java framework for stochastic simulation and evaluation of reaction networks
Source: BMC Bioinformatics. 2008 Aug 29;9:356. doi: 10.1186/1471-2105-9-356 (PMC2553347; doi:10.1186/1471-2105-9-356)
Supplement: Additional file 1 — FERN distribution, Version 1.3. This archive contains the FERN source code and binaries as well as documentation and example models in FernML and SBML. [file 1471-2105-9-356-S1.zip › fern/doc/javadoc/fern/simulation/Simulator.FireType.html]

Simulator.FireType


---


|  |  |  |
| --- | --- | --- |
| **Overview** \| **Package** \| **Class** \| **Use** \| **Tree** \| **Deprecated** \| **Index** \| **Help** | |  |
| **PREV CLASS**   NEXT CLASS | **FRAMES**    **NO FRAMES**     **All Classes** |  |
| SUMMARY: NESTED \| ENUM CONSTANTS \| FIELD \| METHOD | DETAIL: ENUM CONSTANTS \| FIELD \| METHOD |  |


---


## fern.simulation Enum Simulator.FireType

```
java.lang.Object
  java.lang.Enum<Simulator.FireType>
      fern.simulation.Simulator.FireType
```

**All Implemented Interfaces:**: Serializable, Comparable<Simulator.FireType>

**Enclosing class:**: Simulator

---

``` public static enum Simulator.FireType extends Enum<Simulator.FireType> ```

Defines different types of a firing for reactions.

**Author:**
:   Florian Erhard

---

**Enum Constant Summary**

`GibsonBruck`

`GillespieEnhanced`

`GillespieSimple`

`TauLeapCritical`

`TauLeapNonCritical`


| **Method Summary** | |
| --- | --- |
| `static Simulator.FireType` | `valueOf(String name)`             Returns the enum constant of this type with the specified name. |
| `static Simulator.FireType[]` | `values()`             Returns an array containing the constants of this enum type, in the order they are declared. |

**Methods inherited from class java.lang.Enum**

`clone, compareTo, equals, finalize, getDeclaringClass, hashCode, name, ordinal, toString, valueOf`

**Methods inherited from class java.lang.Object**

`getClass, notify, notifyAll, wait, wait, wait`

**Enum Constant Detail**

### GillespieSimple

```
public static final Simulator.FireType GillespieSimple
```

---


### GillespieEnhanced

```
public static final Simulator.FireType GillespieEnhanced
```

---


### GibsonBruck

```
public static final Simulator.FireType GibsonBruck
```

---


### TauLeapCritical

```
public static final Simulator.FireType TauLeapCritical
```

---


### TauLeapNonCritical

```
public static final Simulator.FireType TauLeapNonCritical
```


**Method Detail**

### values

```
public static Simulator.FireType[] values()
```

:   Returns an array containing the constants of this enum type, in
    the order they are declared. This method may be used to iterate
    over the constants as follows:

    ```
    for (Simulator.FireType c : Simulator.FireType.values())
        System.out.println(c);
    ```

    :   **Returns:**: an array containing the constants of this enum type, in the order they are declared

---


### valueOf

```
public static Simulator.FireType valueOf(String name)
```

:   Returns the enum constant of this type with the specified name.
    The string must match *exactly* an identifier used to declare an
    enum constant in this type. (Extraneous whitespace characters are
    not permitted.)

    :   **Parameters:**: `name` - the name of the enum constant to be returned. **Returns:**: the enum constant with the specified name **Throws:**: `IllegalArgumentException` - if this enum type has no constant with the specified name: `NullPointerException` - if the argument is null


---


|  |  |  |
| --- | --- | --- |
| **Overview** \| **Package** \| **Class** \| **Use** \| **Tree** \| **Deprecated** \| **Index** \| **Help** | |  |
| **PREV CLASS**   NEXT CLASS | **FRAMES**    **NO FRAMES**     **All Classes** |  |
| SUMMARY: NESTED \| ENUM CONSTANTS \| FIELD \| METHOD | DETAIL: ENUM CONSTANTS \| FIELD \| METHOD |  |


---
